# Phylogenetic Determinants Behind the Ecological Traits of Relic Tree Family Juglandaceae, Their Root-Associated Symbionts, and Response to Climate Change

**DOI:** 10.3390/ijms26146866

**Published:** 2025-07-17

**Authors:** Robin Wilgan

**Affiliations:** Institute of Dendrology, Polish Academy of Science, Parkowa 5, 62-035 Kórnik, Poland; rwilgan@man.poznan.pl

**Keywords:** phylogenetic traits, relic trees, climate adaptation, root symbiosis, mycorrhiza

## Abstract

Dual mycorrhizal symbiosis, i.e., the association with both arbuscular and ectomycorrhizal fungal symbionts, is an ambiguous phenomenon concurrently considered as common among various genetic lineages of trees and a result of bias in data analyses. Recent studies have shown that the ability to form dual mycorrhizal associations is a distinguishing factor for the continental-scale invasion of alien tree species. However, the phylogenetic mechanisms that drive it remain unclear. In this study, all the evidence on root-associated symbionts of Juglandaceae from South and North America, Asia, and Europe was combined and re-analysed following current knowledge and modern molecular-based identification methods. The Juglandaceae family was revealed to represent a specific pattern of symbiotic interactions that are rare among deciduous trees and absent among conifers. Closely related phylogenetic lineages of trees usually share the same type of symbiosis, but Juglandaceae contains several possible ones concurrently. The hyperdiversity of root symbionts of Juglandaceae, unlike other tree families, was concurrently found in Central and North America, Asia, and Europe, indicating its phylogenetic determinants, which endured geographical isolation. However, for many Juglandaceae, including the invasive *Juglans* and *Pterocarya* species, this was never studied or was studied only with outdated methods. Further molecular research on root symbionts of Juglandaceae, providing long sequences and high taxonomic resolutions, is required to explain their ecological roles.

## 1. Introduction

**Figure 1:**
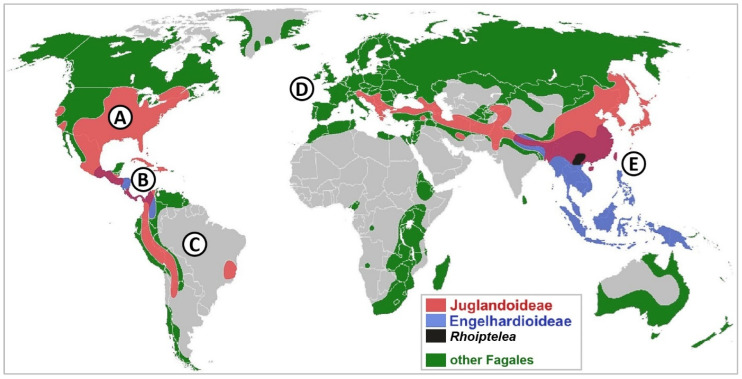
Global distribution of Juglandaceae lineages /juglandoideae (purple; A–E; *Juglans*, *Carya*, *Pterocarya*), /engelhardioideae (blue; B, E; *Alfaroa*, *Engelhardia*, *Oreomunnea*), *Rhoiptelea* (black), and other lineages of Fagales. Concurrent distribution overlap in Mesoamerica (B) and East Asia (E).

The occurrence, role, and importance of dual symbiosis are insufficiently recognised for Juglandaceae. Despite their status as relic family [1], some Juglandaceae species are classified as invasive, such as *Pterocarya fraxinifolia* [2] and *Juglans regia* [3]. The areas of suitable niches for these species will increase [2,3]. Extant Juglandaceae are mainly distributed in Eastern Asia and North and South America, mostly in temperate and montane climates (Figure 1). Juglandaceae originated in North America and Europe during the early Eocene, and its widespread dispersal occurred 13–26 Mya [1]. Following this, Juglandaceae ranges shifted towards the southern regions of Asia and Mesoamerica, but all except *Juglans* are extinct in Europe [4]. Regrettably, a global-scale study on Juglandaceae ecology omitted root symbionts [1], despite mycorrhizal fungi being obligatory symbionts, necessary for the proper functioning of trees, and thereby forest ecosystems [5].

In temperate forests, ectomycorrhizal (ECM) fungi, associated with ECM trees, are the dominant group of root symbionts [6,7,8], but arbuscular mycorrhizal (AM) trees are rather rare [9,10,11]. The diversity and functions of root-associated symbionts are shaped by biogeography, local adaptations, and biotic interactions, which act at different intensities and scales [12,13,14]. In forest soil, the taxonomic and functional structure of mycorrhizal assemblages are driven by abiotic soil factors, such as moisture, pH, and C/N ratio, and by biotic factors, like vegetation structure, plant–plant interactions [6,15,16,17], the taxonomic identity of trees [18,19,20], and the mycorrhizal status (AM/ECM) of trees growing in their surrounding [16,19]. Ectomycorrhizal fungi exhibit a higher ratio of specific C and N enzyme activities than arbuscular fungi, which influences soil carbon and nitrogen cycles, and results in C:N imbalance [16]. Thus, the potential to form symbiosis with ECM and AM fungi has contributed to the resistance of Juglandaceae in unfavourable conditions and their survival 13 Mya after large-scale distribution in the Northern Hemisphere [1,4].

Dual mycorrhizal symbiosis, i.e., the association of tree roots with both arbuscular mycorrhizal and ectomycorrhizal symbionts, is an ambiguous phenomenon and is concurrently considered both common for various tree lineages [21] and as a result of bias in data analyses [22]. Some deciduous tree genera, such as *Eucalyptus* (Myrtaceae), *Alnus* (Betulaceae), *Salix* and *Populus* (Salixaceae), and *Acacia* sensu lato (Mimosoideae and Fabaceae), are well-known for their ability to form dual mycorrhizal associations [21]. Dual mycorrhizal symbiosis is common among trees in tropical and subtropical regions [21,22].

The prevalence of dual mycorrhizal symbiosis among trees in temperate forests is under debate [21,22]. The arbuscular colonisation of ECM-dominated trees’ roots was found in North American tree lineages such as red oaks (*Quercus* sect. *Lobatae* and Fagaceae) [23] and hickories (*Carya* spp. and Juglandaceae) [24,25], but not for widespread European tree species of Fagaceae (*Fagus sylvatica*, *Q. robur*, and *Q. petraea*), nor Pinaceae (*Pinus sylvestris* and *Picea abies*). Species niche modelling for tree species has shown that ECM tree species, including the mentioned Fagaceae and Pinaceae tree families, will lose optimal climatic niches, while AM tree species, such as the native *Fraxinus excelsior* and the invasive alien tree *Robinia pseudoacacia*, will be the best survivors of climate change [26]. Concurrently, *Robinia* poses the most significant and largest negative impact on native ECM fungi [19].

At the global scale, dual mycorrhizal symbiosis has been revealed as a crucial factor in tree species adaptations to climatic and environmental changes [21,27], their acclimatisation to new habitats situated outside their native ranges [27,28], and finally, continental-scale invasions of alien tree species [27]. Metanalyses of 89 tree genera and 32 tree families have shown that AM symbiosis dominates tree roots in colder climate conditions, in extreme water conditions, such as both drought and flood, and nutrient-poor soils [21]. In addition, the share of AM fungi inside the roots of *Populus* is a few times higher in deep soils (20–30 m) than at the soil surface (0–10 cm) [29]. Depth sampling of tree roots is rare. Nearly 100% of research papers on ECM and/or AM fungi involve soil samples taken at the 0–15 cm depth, which is an additional factor that would bias the results [21].

On the contrary, a part of the arguments against the widespread occurrence of dual mycorrhizal symbiosis [22] are justifiable. Old research papers on dual mycorrhizal associations are outdated and, thus, untrustworthy and probably biased [22]. Outdated studies do not prove the identity of putative ECM fungi and tree species based on DNA sequences, while molecular methods of species identification are indispensable by modern research standards, to exclude misdiagnosis of non-mycorrhizal structures as ECM roots [22]. For instance, saprotrophic fungi *Mycena* and *Entoloma* form pseudo-ectomycorrhizal structures with a fungal mantle, but no Hartig net. Those structures pose no ability to transfer substances between fungal and tree partners. In addition, some Sapindaceae (*Acer* and *Aesculus*) and Rosaceae trees (e.g., *Sorbus*, *Prunus*, and *Crataegus*) form complex root anatomy. These root structures can be misinterpreted as ECM roots if research is based on the morphological assessment only [22]. However, both ECM roots with Hartig net and AM root colonization were found inside *P. serotina* roots and evidenced by molecular identification of both ECM fungal and tree partners, and the microscopic identification of Hartig net [30]. The ECM root colonisation was highly varied between six study locations, from 0–2% to 40–60% [30], which indicates that a high number of independent study sites is needed to determine the occurrence of dual mycorrhizal symbiosis with confidence.

Relying on the above data, it is assumed that the Juglandaceae family represents a specific pattern of dual mycorrhizal symbiotic interactions, which is rare among dominant deciduous trees and absent among conifers (Pinales). Closely related phylogenetic lineages of trees usually share the same type of mycorrhizal symbiosis [31], but Juglandaceae contains both AM-dominated (*Juglans*, *Pterocarya*) and ECM-dominated genera (*Carya*, *Oreomunnea*), and concurrently, hyperdiversity of ECM symbionts occurred simultaneously in a single location [18,32]. Therefore, it is assumed that patterns of root-associated symbiosis formed by Juglandaceae represent phylogenetic-related determinants. The methodological limitations in the detectability of AM and ECM symbionts [33], and their preference for different habitats, climatic conditions, and soil depth [21,28,29,30] were assessed. Finally, it was assumed that the dual mycorrhizal symbiosis of Juglandaceae is undetected, rather than actually absent, according to the conclusion “the absence of evidence is not the evidence of absence”, proposed for the first time by Irish author and historian William Wright in 1888 [34]. It was carried out because Juglandaceae, considered AM trees, are studied using methods dedicated to detecting AM fungi [35,36,37,38,39,40,41] and those considered ECM trees using methods for ECM fungi [42,43,44,45,46]. To summarise, four hypotheses have been formulated as follows:

1. Juglandaceae represent a specific pattern of dual mycorrhizal symbiotic interactions.
2. Traits of root symbiosis of Juglandaceae reflect phylogenetic-related determinants.
3. Dual mycorrhizal associations are undetected, not absent, among Juglandaceae trees.
4. Dual symbiosis contributes to Juglandaceae resistance to unfavorable environments.

## 2. Results

### 2.1. Dual Mycorrhizal Symbiosis Among Juglandaceae Genera

Based on the molecular identification of ECM root tips, dual mycorrhizal colonisation was confirmed for AM-dominated *Juglans regia* associated with *Lactarius sanguifluus* [47]. In addition, a few ECM-only Agaricales genera, *Cortinarius*, *Hymenogaster*, and *Hebeloma* [48], were found inside *J. regia* plantations, where ECM tree species were absent [49]. Root symbionts of other Juglandaceae genera, which are considered AM trees, are either represented by a few studies on AM fungi only (*Cyclocarya* [50,51]) or no studies on root symbionts (*Pterocarya*, *Platycarya*; Table 1). Thus, the presence of dual mycorrhizal symbiosis, i.e., both AM and ECM associations, in the roots of these trees cannot be determined.

Structures formed by arbuscular mycorrhizal fungi were found in the roots of *Carya* seedlings (*C. laciniosa* and *C. cordiformis*) [25] and mature (40 yo) *C. illinoinensis* in pecan orchards [24]. An experimental study has shown that the AM fungus *Rhizophagus irregularis* efficiently colonises *C. illinoinensis* roots and mitigates restricted water supply [52].

Arbuscular fungi were slightly, if even, present [57] inside the roots of ECM-dominated tree genera *Engelhardtia* (aka *Alfaropsis*) and *Oreomunnea* [32,43,57,58]. However, the Glomeraceae family formed by arbuscular mycorrhizal fungi represented about 10% of all root-associated fungi of *Engelhardtia* in Hainan and Guangdong provinces in southeast China (Asia) and 1–2% of in roots of *Oreomunne* in Mexico and Panama (Mesoamerica).

### 2.2. Diversity and Habitat Patterns Among Root Symbionts of Juglandaceae

The root-associated symbionts of Juglandaceae formed 741 symbiotic relationships with 90 other plant species. The 721 relationships formed by 74 tree species were taken into the analyses. Data for other plants (20 relationships) were mostly represented by ericoid shrubs (Ericaceae) and orchids (Orchidaceae), which form orchid and ericoid mycorrhizal types. Some tree-associated ectomycorrhizal fungal species can also endophytically colonise the roots of non-ectomycorrhizal herbaceous plants [59]. Therefore, data on herbaceous plants and ericoid shrubs are excluded from further analyses.

**Figure 2:**
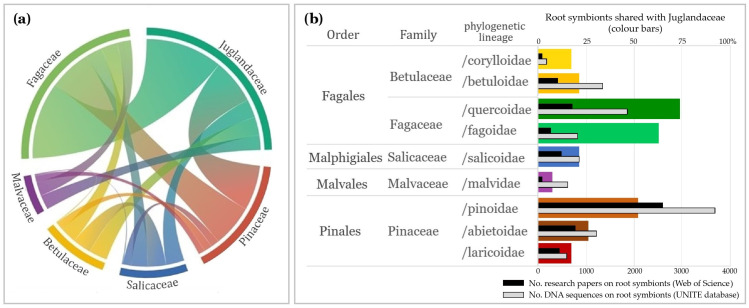
Relationships between root symbionts of Juglandaceae shared with other tree families (**a**) and other tree lineages (**b**), based on 721 relations with 74 tree species, confirmed by molecular data, on the background of research papers and DNA sequences for trees’ root-associated symbionts.

Fagaceae (Fagales) formed 56% of relationships and 88% of tested ECM fungal taxa were associated with *Quercus* (74%) and/or *Fagus* (63% of ECM taxa). Pinaceae (Pinales) formed 25% of relationships, and 52% of ECM fungal taxa were associated with *Pinus* (32%) and/or *Picea* (33%). Betulaceae (Fagales), Salicaceae (Malpighiales), and Malvaceae (Malvales) formed 3–7% of relationships, but with 17–21% of root symbionts (Figure 2).

In addition, the percentage of shared symbionts was shown on the background of the number of research papers (Web of Science database) and DNA sequences on tree root-associated symbionts (UNITE database) to avoid the potential bias due to a varied number of studies, so sequences on root symbionts formed by the main tree lineages. The numbers of shared symbionts were preserved with similar values within tree families (colour bars), even if the number of studies and sequences largely differ (black/gray bars) (Figure 2).

**Figure 3:**
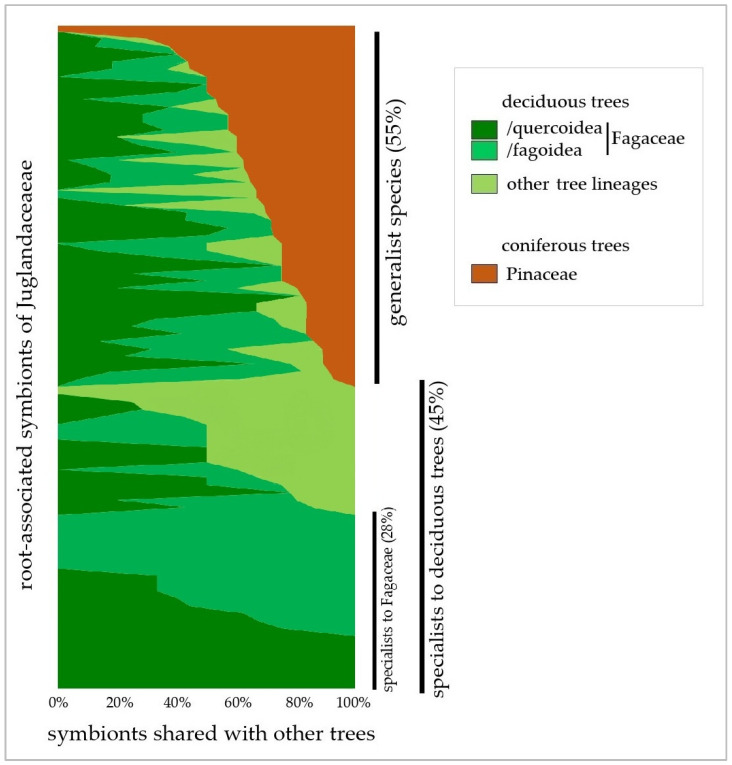
Root symbionts of Juglandaceae: generalists associated with both deciduous and coniferous trees (55%), and specialists to deciduous trees (45%), including Fagaceae-associated ones (28%).

The 721 relationships formed by root symbionts of Juglandaceae were analysed based on the phylogenetic identity of trees. Symbionts were gathered into three groups: specialists associated with Fagaceae (/quercoidea and /fagoidea; ~28%), specialists associated with deciduous trees only (~45%, including Fagaceae-specific symbionts), and generalists that form symbiosis with both deciduous trees and conifers (Pinales) (Figure 3).

**Figure 4:**
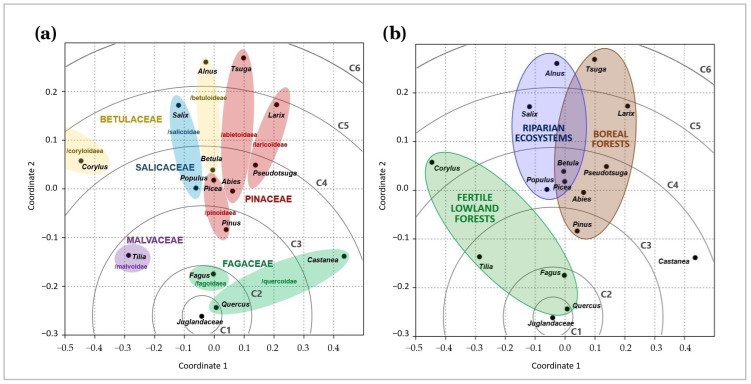
Non-metric multidimensional scaling (NMDS) plots visualising relations between root-associated symbionts of Julgandaceae, their associations with other lineages of trees (**a**), and their main habitats (**b**). Carpinus (/coryloidae, C6, situated at left to Corylus) was an outlier.

The NMDS has also shown that *Quercus* and *Fagus* (Fagaceae) are most closely related (C1 and C2, respectively) to Juglandaceae, while *Alnus* (/betuloidae) and *Tsuga* (/abietoideae) were slightly associated (Figure 4). Both closer and further groups of tree genera were shown by both the phylogenetic lineage approach and the main forest habitat type approach. The observed patterns in similarity and dissimilarity among root-associated symbionts were partly explained by both the phylogenetic identity of trees (NPMANOVA F = 1.25, *p* = 0.031; Figure 4a) and habitat type (NPMANOVA F = 1.29, *p* = 0.029; Figure 4b).

## 3. Discussion

### 3.1. Global Distribution of Dual Mycorrhizal Trees and Mycorrhizal Association Types

Symbiotic associations between plant roots and soil microorganisms turned out to be a crucial factor in acclimation, survival, and range expansion of trees at a global scale [27,60,61,62,63,64,65,66]. Ongoing climate changes lead to the northward shift in the optimal climatic niches for both forest tree species [67,68] and root-associated fungal symbionts of these trees [69,70]. The global-scale distribution of tree root-associated symbiosis shows latitude and altitude patterns. Boreal, temperate, continental, and montane forests are dominated by ECM trees, but AM-associated trees are rarely, if ever, present there. On the contrary, in tropical and subtropical forests, 50–70% of trees form AM symbioses [71], even if ECM trees there can be underestimated [56]. Those differences are rooted in the individual features of both groups of symbionts. Studies on dual mycorrhizal tree genera *Alnus* and *Populus* revealed that AM fungi are reduced in lower soil temperatures, while ECM fungi are not sensitive to this factor. In turn, ECM colonisation on tree roots significantly decreases with drought, in favour of the increasing formation of arbuscular structures, and decreases with poor oxygen availability in flooded soil because anaerobic conditions are unfavourable for ECM fungi [72,73]. In general, harsh habitat conditions such as drought and flooding temporarily favour the dominance of AM symbionts [21,27,74,75]. Woodland ecosystems with high fluctuations of environmental conditions during the year, such as seasonal flooding and drought, are inhabited by dual mycorrhizal trees characterised by their ability to switch between arbuscular and ectomycorrhizal associations, such as drought-resistant *Acacia* and *Eucalyptus*, and flood-resistant *Alnus*, *Salix*, and *Populus* [27].

The native range of Juglandaceae covers cold climates in northern USA, Canada, northern China, mountain regions in the Andes, warm tropical and subtropical climates in Mesoamerica and Asia (Figure 1), and riparian forests [2,76]. The large scale of habitat types and climates raises a question: How widespread is dual symbiosis among Juglandaceae? The evolutionary history for mycorrhizal symbiosis shows that mycorrhizal associations formed by coniferous trees are evolutionary old relationships, shared by all members of the ECM-only *Pinales* order, AM-only Araucariales, and Cupressales [31].

On the other hand, deciduous trees exhibit mixed types of mycorrhizal associations. Individual lineages of ECM trees are usually situated within AM-dominated orders, like ECM genus *Tilia* in AM-dominated Malvales, ECM(+AM) genera *Populus* and *Salix* [29] in AM-dominated Malphigiales, or ECM(+AM) *Acacia* sensu lato in AM-dominated Fabales. Only the Fagales order is considered an ECM-dominated lineage of deciduous trees [31,77]. However, a few lineages of Fagales, like *Alnus* (Betulaceae), *Corylus* (Betulaceae), and *Casuarina* (Casuarinaceae), form dual symbioses [31,77,78]. Juglandaceae, sister clade to Betulaceae [79], so far was considered to be composed of ECM-only (engelhardioideae and *Carya* [18,42,56]), and AM-only trees (/juglandoideae except *Carya*) [35,36,37,38,39,40,41,50,51].

**Figure 5:**
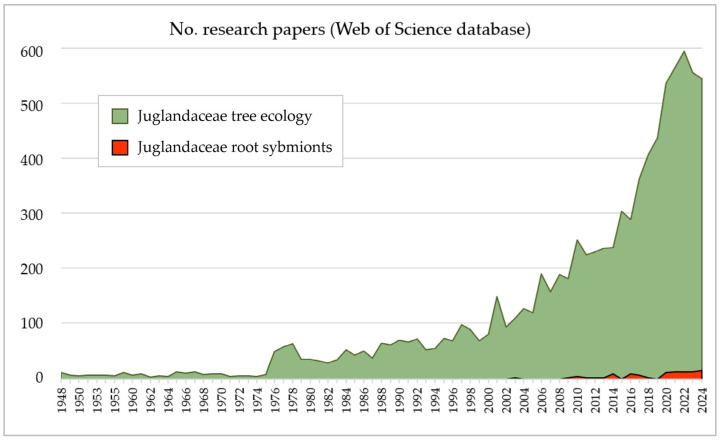
The number of research papers on the ecology of Juglandaceae tree species and their root-associated symbionts published over time.

Therefore, in this study, data on the root symbiotic associations of the relic tree family Juglandaceae were collected and carefully re-analysed against the background of methodological features typical for various molecular methods and their individual limitations. In general, only 0.6% of all research papers on the ecology of Juglandaceae contained data on their root symbionts (Figure 5, Table 1). Root-associated symbionts of 67% of Juglandaceae genera were studied once or never, and >80% of Juglandaceae species have never been studied. Juglandaceae, considered as AM trees, are studied using methods dedicated to AM fungi [35,36,37,38,39,40,41], and those considered ECM trees using methods for ECM fungi [42,43,44,45,46]. This approach, along with the ability of dual mycorrhizal trees can switch between one and another type of symbiotic association [28,29,30], makes dual mycorrhizal associations difficult to find [21]. In view of the above, dual mycorrhizal associations among Juglandaceae tree species should be considered as undetected, rather than absent.

### 3.2. The History of Research on Dual Mycorrhizal Symbiosis Among Juglandaceae

The first evidence of the dual mycorrhizal symbiosis of ECM-dominated Juglandaceae trees was published in 2016 (*Carya* seedlings formed AM symbioses after inoculation by AM fungi under experimental conditions [52]) and then confirmed by two independent studies carried out in the field by research teams in Europe (ECM and AM associations of *Carya* seedlings in botanical garden in Poland; June 2018 [25]) and South America (AM associations of mature *Carya* trees in pecan orchard in Argentina; September 2018 [24]). Additional indirect evidence was provided by a study on root-associated fungi of ECM-dominated genera from subtropical regions: *Engelhardtia* (aka *Alfaropsis*) and *Oreomunnea*. Root-associated assemblages of these trees are dominated by ECM fungi (62–78% of all fungal reads), but AM fungal family Glomeraceae formed 6–10% of fungal reads for *Engelhardtia* and 1–2% for *Oreomunnea* (Figure 3 in [57]). The arbuscular fungal guild was undetected, so not shown in the results (Figure 2 in [57]), because the species-level identification of AM fungi requires the application of AM-specific starters, such as NS31 and AML2 primers for the SSU region of fungal rDNA [33]. Corrales et al. (2021) used standard starters ITS1F and ITS2, widely used in studies on ECM fungi [18,19,80,81] and soil/root mycobiomes [8,82,83] but insufficient to provide the species-level identification of AM fungi, especially at short sequence lengths, as are provided by high-throughput sequencing methods [84]. As a result, the AM fungi and AM guild are not listed (Figure 2 in [57]), even if the AM fungal family Glomeraceae was detected (Figure 3 in [57]).

The share of arbuscular fungal family Glomeraceae inside ECM-dominated tree genera *Engelhardtia* and *Oreomunnea* was highly varied, from 6% and 10% for root-associated fungi in Hainan and Guangdong provinces in China (*Engelhardtia*) to <2% in Panama and Mexico in Mesoamerica (*Oreomunnea*) [57]. Stands in China are situated in a temperate climate (Köppen Cwa/Cfa, altitude 550–850), while those in Mesoamerica are in tropical montane forest (Köppen climates Am/Af, altitude 1200–1600). Additionally, Guangdong and Hainan forests are dominated by AM trees [57]. The cumulative effect of climates, higher altitude, and dominance of AM trees, so AM propagules, explains the higher share of the AM family Glomeraceae in Juglandaceae roots in China than in Mesoamerica.

The first evidence of dual mycorrhizal symbiosis among AM-dominated Juglandaceae trees was published in 2013, when the ECM fungus *Lactarius sanguifluus* was proved to form symbioses with the roots of AM-dominated *Juglans regia* in the Galyat mountains in Pakistan [47]. Indirect evidence of dual mycorrhizal symbiosis was also shown by the presence of ECM fungi *Cortinarius*, *Hymenogaster*, and *Hebeloma* inside monoculture plantations of *J. regia* in Caledonia, Michigan, USA [49]. The mentioned fungal genera are obligatory ectomycorrhizal [48], which means that they require ECM symbiosis with trees for growth and functioning in the forest ecosystem [48]. The occurrence of obligatory ECM fungi inside the monoculture plantations of *Juglans regia,* where other tree species were absent [48], indicates the potential occurrence of symbiotic associations between these ECM fungi and *Juglans* roots [48]. Other genera of Juglandaceae, *Pterocarya*, *Cyclocarya*, and *Platycarya*, are represented by no study on root-associated symbiosis, or by a few studies on AM symbiosis only [50,51]. Therefore, the occurrence of dual mycorrhizal symbiosis among them cannot be determined nor excluded.

### 3.3. Tree Phylogeny and Forest Habitat as Main Factors Shaping Root Symbiosis of Juglandaceae

The highest part of the root symbionts of Juglandaceae (Fagales) was shared with the closely related tree family Fagaceae (Fagales) (Figure 2, Figure 3 and Figure 4). The climatic optimum for Juglandaceae occurred 50 Mya [79], and then, their optimal climatic niches subsequently declined. Juglandaceae became extinct in Europe ~2 Mya [2,4], except for *Juglans regia*. However, *J. regia* is native to the Caucasus and Central Asia but is now considered an alien species in Europe, where this tree was widely planted due to its edible nuts [3,85].

Using all available data in the UNITE database, 75% of root-associated symbionts of Juglandaceae were revealed to be shared between Juglandaceae and closely related tree lineage /quercoidae (Figure 2 and Figure 3). In their native forest habitats, Juglandaceae trees *Engelhardtia*, *Oreomunnea*, *Carya*, and *Juglands* commonly occur with /quercoidae trees, most often co-creating forest ecosystems with *Quercus* species [46,57,86,87], but also *Cyclobalanopsis*, *Castanopsis*, and *Lithocarpus* in southern Asia [32]. *Quercus* is an important forest tree genus with high species diversity in Mesoamerica, the USA, China, and Southeast Asia [88], the very same diversity hotspots as determined for Juglandaceae [1].

The co-evolution of Fagales and their root-associated mycorrhizal symbionts dates back to 100 Mya [31], while considerable diversification within phylogenetic lineages of mycorrhizal fungi has started in the late Cretaceous and Palaeocene, about 50 Mya [89,90]. Indications are that, despite the geographical isolation, root symbionts of Fagales are compatible with geographically separated lineages of trees. For instance, North American red oak (*Q. rubra*, *Quercus* sect. *Lobatae*) growing under forest conditions in Europe, outside its native continent, easily entered symbiosis with a few hundred local root symbionts typical for European oaks (*Quercus* sect. *Quercus*) [19,20,91]. A similar pattern was found for *Carya* trees, which easily enter associations with local oak symbionts [18,25,92].

Hyperdiversity of the root symbionts of Juglandaceae, which was similar or higher than the diversity of neighbouring forest ecosystems with multiple ECM tree species, was found in Europe [18,25], and simultaneously, high diversity of symbionts at the species, genus, and family levels was reported in Mesoamerica, USA, and southern Asia [32,46].

The ability of Juglandaceae to form dual mycorrhizal associations was independently found in Europe, America, and Asia [24,25,47,52], which indicates the occurrence of phylogenetic-related determinants that endured geographical isolation. However, phylogenetic distance explained only a part of the dissimilarity shown by NMDS. The main type of forest habitat inhabited by various trees was shown to be a more consistent predictor of the observed dissimilarity (Figure 4b) than phylogenetic distance between trees (Figure 4a), even if both phylogeny and habitat presented similar significance and F-values.

Interestingly, an experimental study on entire root systems of tree seedlings grown under controlled conditions in five forest nurseries in the field has already shown that forest habitat is a more important predictor of the similarity between tree root symbionts than phylogenetic distance [93]. Two trees of large phylogenetic distances, *Carpinus* (Betulaceae, Fagales) and *Tilia* (Malvaceae, Malvales), shared 64.3% of root symbionts, while closely related trees, *Carpinus* and *Betula* (Betulaceae, Fagales), only 38.7%. *Carpinus* and *Tilia* inhabit fertile lowland forests, while *Betula* inhabits nitrogen-poor boreal and mixed coniferous forests [6]. The NMDS has shown that the main forest habitats (fertile forests, riparian ecosystems, and boreal/montane forests) most clearly explain observed dissimilarity. A study in Alpine habitats showed that altitude, not phylogenetic distance between plant species, determines the composition of root-associated symbionts [94]. A study on European *Abies alba* and North-American *A. grandis* even showed that lowland and mountain regions determine the differences in trees’ symbionts more than trees’ origins [7].

### 3.4. Relevance of Dual Mycorrhiza for Juglandaceae Trees and Directions for Further Research

The ability to form both AM and ECM symbioses provides tree species with additional advantages in changing environments. Climate change leads to a continental-scale northward shift in the optimal niches for forest tree species [67,68], including *Carya*, *Pterocarya*, and *Juglans* [2,3,70]. The ability to form ECM symbioses through AM-dominated tree species provides trees with better adaptation to cold continental, montane, and boreal climates, where ECM fungi form almost all the mycorrhizal interactions of tree roots [71]. Similarly, the ability to form AM symbioses through ECM-dominated trees provides trees with benefits under high temperatures, drought, and low oxygen content in flooded soil.

Further molecular research on both AM and ECM root symbionts of Juglandaceae trees, supported by the platform providing long DNA sequences and high taxonomic resolutions, is necessary to explain the role of the observed patterns of root symbiosis in the climate adaptation and future range expansion of Juglandaceae species. Further research should be conducted using various habitat types and geographical locations to detect potential occurrences of dual mycorrhizal associations. The last study on root-associated fungi of AM-dominated *Prunus serotina* in France revealed extremely high variability in the share of ECM symbionts between the stands; ECM fungi reached from 0 to 2% of all root-associated fungi on three locations, but up to 45–60% on two other locations of *P. serotina* [30]. These results highlighted the essential role of various habitats and numerous geographical locations in the study on dual mycorrhizal symbiosis. *Prunus serotina*, one of the five most invasive tree species in Europe [95], which was previously considered as an AM tree only [30], had a rather low negative impact on ECM fungi, even in the forest ecosystems with a high density (>70%) of *P. serotina* trees [19], which may result from trees’ ability to maintain ECM associations, besides the main AM symbiosis [30].

Juglandaceae trees, such as AM-dominated *Juglans regia* and *Pterocarya fraxinifolia*, are considered invasive and potentially invasive in the future, with their invasiveness increasing along with climate change [2,3]. Similarly, *Carya illinoinensis*, a widely cultivated tree used in the co-production of pecan nuts and black truffles [42,53,54,55], will increase in the range of suitable niches [70]. The effects of global warming have already been observed [96] and are expected to accelerate rapidly in the future [97,98]. Both *J. regia* and *C. illinoinensis* were proven to form dual mycorrhizal associations [24,47,52]. The influence of dual mycorrhizal associations on the further expansion of Juglandaceae trees is unknown. However, all evidence on the ecological advantages of dual mycorrhizal associations for alien tree species indicates that the beneficial role of dual associations for Juglandaceae adaptations would be expected [27]. Further comprehensive studies are needed to obtain essential data to determine in which habitats Juglandaceae species could be present and potentially invasive, and wherein they can have a significant impact on native biota.

## 4. Materials and Methods

**Figure 6:**
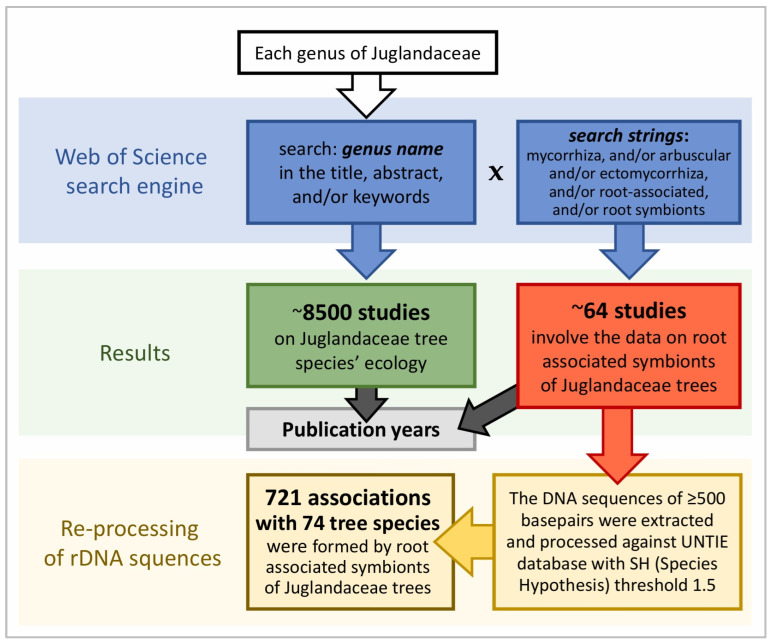
Flow diagram of the study search and data re-processing.

To analyse the dual mycorrhizal symbiotic interactions among Juglandaceae trees, all the evidence on root-associated symbionts of tree genera from South and North Americas, Asia, and Europe was collected. Five main regions were determined: North America, with a temperate climate (A); the tropical regions in Mesoamerica (B); the montane region in Andes (C); the temperate climate in Europe (D); and the tropical and subtropical climates in southern Asia (E) (Figure 1). Out of 8500 studies on Juglandaceae trees ecology, 64 studies (~0.6% of all) concerned mycorrhizal symbionts associated with Juglandaceae trees (Table 1, Figure 5). Then, the 64 research papers were analysed to assess the potential ability of trees to form dual mycorrhizal symbiosis, i.e., ECM colonisation on the roots of AM trees, and AM fungi inside the roots of ECM trees. The molecular methods used to identify root symbionts were assessed, including the presence of specific starters necessary to identify arbuscular mycorrhizal fungi and the microscopic identification of the structures they formed [29]. In the next step, molecular datasets were re-analysed using UNTIE v. 9.0. Sequences were assigned to Species Hypotheses (SH) with a threshold 1.5, using the BLAST algorithm [99]. Threshold 1.5 (>98.5 sequence similarity) has been implemented to avoid both taxonomic bias in by-passing the species caused by too low a threshold (i.e., threshold 3, with >97 similarity) and the potential confusion between species diversity and intraspecific variation, caused by too high a threshold (i.e., threshold 0.5–0.0, with 99.5–100% sequence similarity only) [100]. Sequences assigned to Species Hypotheses by the members and associates of the UNTIE Community were used, if available. If not, the sequences were supplemented by SH, assigned automatically by the program. Next, the tree species reported as the tree partners of root-associated fungal species were extracted from the UNITE database and analysed. A flow chart is given in Figure 6. Analyses were prepared using the phylogenetic identity of plant and fungal species—partners of symbiosis, from the UNITE database and the literature [9,31,48,101,102].

To prepare phylogenetic analyses of shared partners among root-associated symbionts of Juglandaceae, data on the Internal Transcribed Spacer (ITS) region of fungal rDNA sequenced from ECM roots (500 base pairs and more; usually results of Sanger sequencing) were applied. Sequences provided by the metabarcoding method (NGS) are too short (<300 base pairs) [103] for species-level identification of ECM fungi. Instead, sequences provided by metabarcoding are grouped in clusters called OTUs (Operational Taxonomic Units) based on the sequences’ similarity. As a result, it cannot be determined that a single OTU represents one ECM fungal species or a few closely related ones. Nevertheless, the list of ECM fungal OTUs represents a summary of genetically different groups. Thus, even if the actual number of ECM fungal species would be higher, which is shown by the ECM fungal sporocarp identification compared to NGS results [104], NGS sequencing is still useful in studies on fungal diversity because it provides valuable data on a higher-level diversity of various of genetic groups within the genus, such as groups of closely related species, clades, and phylogenetic lineages of ECM fungi [9,48].

The aforementioned dataset on the shared tree partners among root symbionts of Juglandaceae trees was used to prepare non-metric multidimensional scaling (NMDS), a distance-based ordination technique, to visualise relations between root symbionts and their tree partners at two levels. One level contained the phylogenetic distance between tree genera, and the second contained the data on the main forest habitat type, inhabited by each tree genus: riparian forests with high water levels, montane forests with low temperatures and short vegetation periods, and lowland forests with moderate temperatures and water levels. The phylogenetic relations between fungal and tree partners shown in the circular diagram were generated using R software v4.2.0 [105] with the package circlise [106], and NMDS ordination using PAST ver. 2.17 (available online: https://palaeo-electronica.org/2001_1/past/pastprog/index.html (accessed on 13 March 2025)).

## 5. Conclusions

To conclude, the cross-sectional analyses of all available data on tree root-associated symbionts of Juglandaceae in South and North America, Europe, and Asia have shown phylogenetic-related determinants and habitat type-oriented patterns behind the ecology of root-associated symbiosis formed by the relic tree family Juglandaceae.

Juglandaceae trees were revealed to form dual mycorrhizal symbiosis, i.e., associations with AM and ECM root symbionts concurrently. This is a specific pattern of symbiotic interactions, rare among deciduous trees and absent among conifers, because closely related phylogenetic lineages of trees usually share one and the same type of mycorrhizal symbiosis. High diversity among root symbionts of Juglandaceae was independently found in the tropics and temperate forests in Mesoamerica, Asia, and Europe, which indicates the phylogenetic-related determinants that endured geographical isolation. Similarities between root symbionts shared by Juglandaceae and other tree lineages were shaped by the main forest habitat type, besides the phylogenetic distance between trees.

Root symbionts of many Juglandaceae, including invasive *Juglans* and *Pterocarya* species, have never been studied or have only been studied with outdated methods. The impact of dual symbiosis on further expansion of these trees is unknown, but evidence on the ecological advantages of dual symbiosis for alien trees indicates its beneficial role [27].

Further extensive molecular studies on both AM and ECM root symbionts of various Juglandaceae tree genera, growing under various environmental conditions, are necessary to explain the distribution of dual mycorrhizal associations among Juglandaceae, and their ecological roles in environmental adaptation, climate resilience, and potential future expansions of Juglandaceae species. Comprehensive research is also needed to obtain essential data to determine in which habitats alien Juglandaceae species can be present and potentially invasive in the future, and where they can significantly impact native biota.

## Figures and Tables

**Table 1:** Number of studies on the ecology of Juglandaceae tree genera, and research papers on their root-associated symbiotic associations (if the number is >10, few recent studies were found).

Juglandaceae		Number of Research Papers		
Phylogenetic Lineage	Genus	Tree Ecology *	Root-Associated Symbionts	
			AM Fungi	ECM Fungi
/juglandoide	*Juglans*	~5000	29 studies (e.g., [35,36,37,38,39,40,41])	2 studies [47,49]
	*Carya*	>2500	3 studies [24,25,52]	10 studies [18,20,25,42,44,45,46,53,54,55]
	*Pterocarya*	~300	-	-
	*Cyclocarya*	~500	2 study [50,51]	-
	*Platycarya*	~100	-	-
/engelhardioidea	*Alfaroa*	~20	-	1 study [56]
	*Engelhardtia* (=*Alfaropsis*)	~60	1 study [57]	1 study [57]
	*Oreomunnea*	~50	1 study [57]	4 studies [32,43,57,58]
outgroup	*Rhoiptelea*	~30	-	-
All Juglandaceae trees		~8500 (100%)	35 (0.4%)	17 (0.2%)

* Tree genus mentioned in the abstract, keywords, and/or publication title.

## Data Availability

All molecular data reprocessed for this study are available in the reference research papers listed in Table 1.
